# Oxidative stress, gene expression and histopathology of cultured gilthead sea bream (*Sparus aurata)* naturally co-infected with *Ergasilus sieboldi* and *Vibrio alginolyticus*

**DOI:** 10.1186/s12917-023-03840-9

**Published:** 2023-12-16

**Authors:** Mahmoud Abou-Okada, Maha M. Rashad, Ghada E. Ali, Shimaa Abdel-Radi, Azza Hassan

**Affiliations:** 1 Department of Aquatic Animal Medicine and Management, Faculty of Veterinary Medicine, Cairo University, Giza, 12211 Egypt; 2 Department of Biochemistry and Chemistry of Nutrition, Faculty of Veterinary Medicine, Cairo University, Giza, 12211 Egypt; 3 Department of Parasitology, Faculty of Veterinary Medicine, Cairo University, Giza, 12211 Egypt; 4 Department of Pathology, Faculty of Veterinary Medicine, Cairo University, Giza, 12211 Egypt

**Keywords:** *Sparus aurata*, *Ergasilus sieboldi*, *Vibrio alginolyticus*, Proinflammatory cytokines, Host defense mechanism, Gill pathology

## Abstract

**Background:**

Parasitic and bacterial co-infections have been associated with increasing fish mortalities and severe economic losses in aquaculture through the past three decades. The aim of this study was to evaluate the oxidative stress, histopathology, and immune gene expression profile of gilthead sea bream (*Sparus aurata*) co-infected with *Ergasilus sieboldi* and *Vibrio alginolyticus*.

**Results:**

*Vibrio alginolyticus* and *Ergasilus sieboldi* were identified using 16 S rRNA and 28 S rRNA sequencing, respectively. The collagenase virulence gene was found in all *Vibrio alginolyticus* isolates, and the multiple antimicrobial resistance index ranged from 0.286 to 0.857. Oxidant-antioxidant parameters in the gills, skin, and muscles of naturally infected fish revealed increased lipid peroxidation levels and a decrease in catalase and glutathione antioxidant activities. Moreover, naturally co-infected gilthead sea bream exhibited substantial up-regulation of *il-1β, tnf-α*, and *cyp1a1*. *Ergasilus sieboldi* encircled gill lamellae with its second antennae, exhibited severe gill architectural deformation with extensive eosinophilic granular cell infiltration. *Vibrio alginolyticus* infection caused skin and muscle necrosis in gilthead sea bream.

**Conclusion:**

This study described some details about the gill, skin and muscle tissue defense mechanisms of gilthead sea bream against *Ergasilus sieboldi* and *Vibrio alginolyticus* co-infections. The prevalence of co-infections was 100%, and no resistant fish were detected. These co-infections imbalance the health status of the fish by hampering the oxidant-antioxidant mechanisms and proinflammatory/inflammatory immune genes to a more detrimental side. Our results suggest that simultaneous screening for bacterial and parasitic pathogens should be considered.

**Supplementary Information:**

The online version contains supplementary material available at 10.1186/s12917-023-03840-9.

## Background

The aquaculture sector has an essential role in supplying more than 50% of fish and fish products to the world population [1]. Several bacterial, viral, and parasitic illnesses have plagued gilthead sea bream (*Sparus aurata*) during the last few decades, posing major challenges to fish production and profitability [2].

Copepods are among the most prevalent destructive ectoparasites of farmed marine fish [3, 4], whereas vibriosis is the most dangerous bacterial infection [5, 6]. *Ergasilus sieboldi* is a common ectoparasitic copepod that infects primarily the gill filaments of fresh, brackish, and seawater fish, while additional microhabitats include the base of the fins, the external surface of the operculum, and the urinary bladder [7, 8]. *Ergasilus sieboldi-*mediated infections are seasonal, with parasite populations peaking during late summer and autumn. They induce respiratory distress, slow growth, sluggish behavior, and increased vulnerability to secondary illnesses such as bacterial infections [9–11].

*Vibrio alginolyticus* is pathogenic to gilthead sea bream through a series of events that begin with adhesion to sea bream mucus, then proliferation, colonization of the underlying epithelium, and finally secretion of hydrolytic enzymes, which may be responsible for the development of ulcers and extensive tissue damage [12, 13]. Various virulence factors encoded by virulence genes have been proposed as key contributors to the pathogenicity of *V. alginolyticus* [14].

Co-infections are infections of a host by two or more genetically distinct pathogens. Each pathogen is responsible for its pathogenic effects and contributes to the overall damage to the host when combined with other pathogens [15, 16]. Parasitic infections increase the risk of secondary bacterial diseases and can act as a vehicle to transmit bacterial pathogens [17]. This synergistic effect has been explained as a result of the stress caused by parasites reducing the resistance of fish to other secondary bacterial infections [18]. Co-infections significantly impact fish health and can potentially change several fish diseases’ progression and severity [19, 20]. Several studies reported parasitic and bacterial co-infections in Nile tilapia (*Oreochromis niloticus*) [21] goldfish (*Carassius auratus*) [22], Atlantic salmon (*Salmo salar*) [23] and flathead grey mullet (*Mugil cephalus*) [24].

The histopathological alteration, oxidative stress response, and immune-related gene expression may contribute to understanding the host response to pathogenic invasion [25, 26]. The innate immune system is the first to respond to infection and disease and does not retain memory of previous responses [27]. It is a crucial factor in resistance to disease [28], comprising the epithelial/mucosal barrier, humoral parameters, and immune cells [27, 29]. Mucus is the first line of defense and consists of mucins, which can be categorized as structural, associated with the plasmalemma, and secreted, which form the outer mucus gel layer [30]. As well as being a physical barrier, glycoprotein components of mucus are active in combating pathogens and parasites [30, 31]. Hyperplasia and hypertrophy of mucous cells have been described in fish with crustacean ectoparasite infections [32].

Parasitic copepods induce damage to the host through feeding or attachment to the skin or other surfaces by means of clawed limbs. On fish body surfaces, attachment can provoke epidermal erosion and necrosis and host responses including fibroblast proliferation, recruitment of immune cells, and increased collagen fibers at site of attachment [33]. Attachment to fish gill induces fusion of secondary lamellae, consequently reducing the respiratory surface of the organ [34]. The means by which *E. sieboldi* attaches and feeds cause considerable gill pathology. The insertion of the copepod antennae deep into gill tissue causes disruption of the gill filaments [32, 33, 35]. Heavy infections by *E. sieboldi* result in severe respiratory problems in fish, especially during warms months [10, 33].

Oxidative burst and the reactive oxygen species (ROS) production is an important defense mechanism in the immune response of aquatic organisms to infections [36]. Assessment of oxidant and antioxidant defense pathways is considered an excellent diagnostic and prognostic tool in aquatic organisms [37]. Glutathione is a non-enzymatic co-factor for glutathione-S-transferase, prevents the deleterious effects of ROS with the production of glutathione disulfide (GSSG), which play a vital role in counteracting oxidative stress and protecting cells from lipid peroxidation, cellular injury, and cellular apoptosis [35, 38]. Moreover, catalase (CAT) is a common antioxidant enzyme that utilizes oxygen and acts as a critical immune-related gene in the teleost, playing an essential role in the immune defense against pathogenic invasion [39, 40].

Immune gene expression profiles are crucial for understanding the molecular pathogenesis and disease development process associated with infections [41–43]. Cytochrome P450 family 1 subfamily A member 1 (*cyp1a1*) is considered the most active xenobiotic-metabolizing enzyme of cytochrome P450 [44], whereas interleukin-1β (*il-1β*) and tumor necrosis factor alpha (*tnf-α*) are classical proinflammatory cytokines [45]. *Il-1β* plays an essential role in coordinating fish responses to infections. It increases the expression of inflammation-related molecules and induces the release of other cytokines [45]. The expression dynamics of immune gene profile, prooxidant-antioxidant biomarker, and pathological changes can help to reveal fish defensive mechanisms against infections [25, 26]. Therefore, this study unlocks the enigmatic causes behind the summer mortalities in cultured gilthead sea bream reared in semi-intensive earthen pond-based marine fish farming and analyses the oxidative stress, histopathology, and immune gene expression profile of gilthead sea bream in response to parasitic and bacterial co-infections.

## Results

### Case history, clinical and parasitological examinations

Based on clinical, parasitological and bacteriological examination, the uninfected control fish were free from macroscopic and microscopic parasites as well as pathogenic bacteria.

**Fig. 1:**
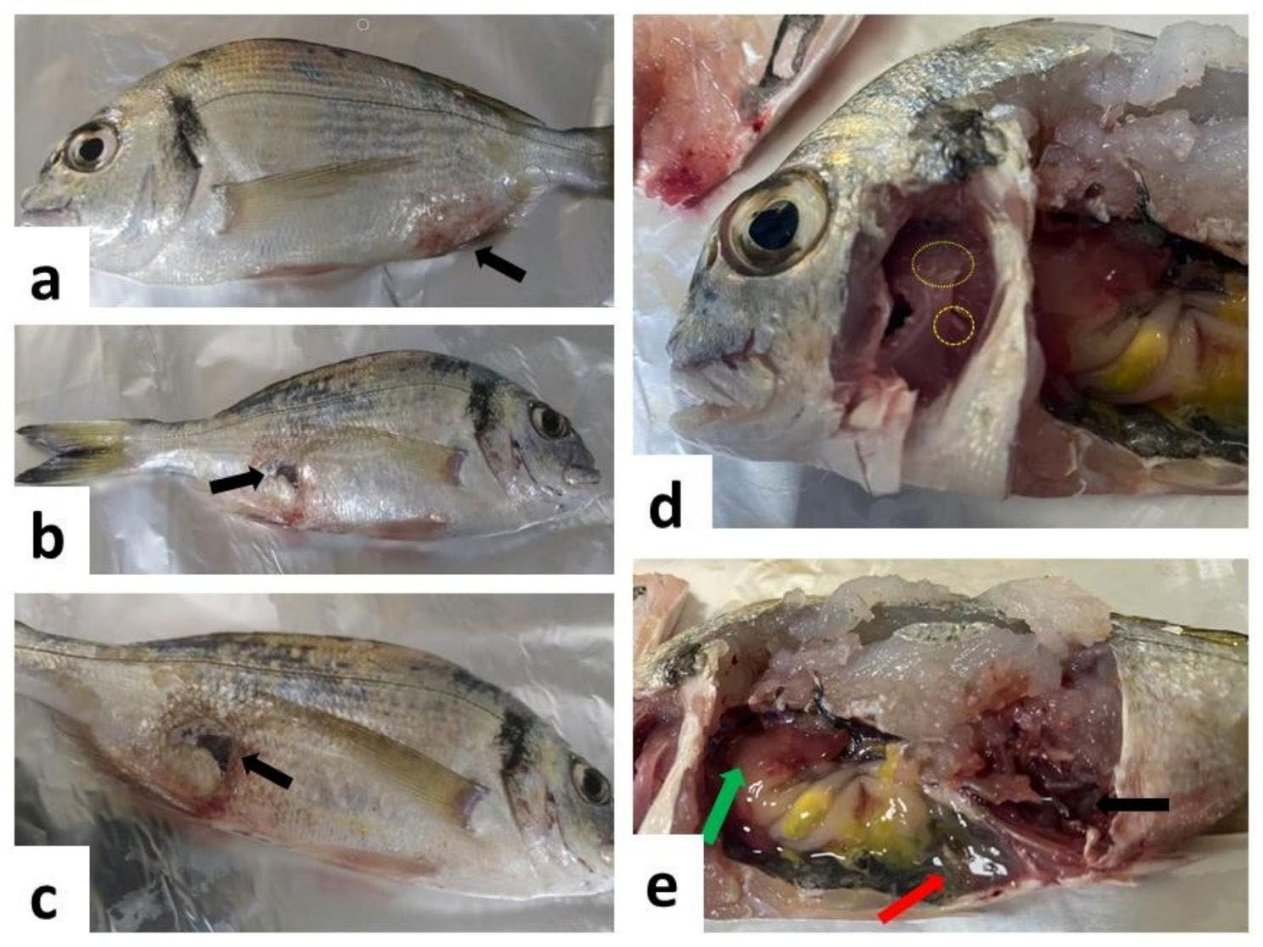
Clinical signs, and postmortem examination of naturally infected gilthead sea bream (*Sparus aurata*), **(a)** detached scales, skin hemorrhage and abrasion (black arrow), **(b)** detached scales, skin hemorrhage, skin and muscle ulceration (black arrow), **(c)** severe skin and muscle ulceration (black arrow), **(d)** Pale gills (dotted yellow circles), **(e)** hemorrhagic muscle (black arrow), pale liver with congested borders (green arrow) and serosanguinous fluids in abdominal cavity (red arrow)

Naturally infected gilthead sea bream showed abnormal swimming behavior, respiratory distress with apparent signs of asphyxia. A clinical examination indicated skin darkening, detached scales, skin hemorrhage and skin abrasion (Fig. 1a) that progressed to skin ulceration (Fig. 1b) and muscle ulceration (Fig. 1c). On the other hand, the infected fish had pale gills (Fig. 1d). Furthermore, the abdominal cavity was filled with serosanguinous fluids, hemorrhagic muscle, and pale liver with hemorrhagic borders (Fig. 1e).

Microscopic examination of gill filaments revealed microscopic parasite. This parasite had a total body mean length of 1.2 ± 0.05 mm (range: 1.1–1.4 mm), pyriform cephalothorax with lateral constriction toward the posterior section and thoracic segments that narrow posteriorly. The parasite had two elongated egg sacs at the end of the adult female body. Moreover, it had two well-developed antennae. Each antenna consisted of coxobasis and three endopodite segments with a large, strong bent claw at the end, responsible for grasping the gills. Based on the previous morphometric characteristics, we confirm that these parasites belong to parasitic copepopds, genus *Ergasilus Nordmann*, 1832 and identified as *Ergasilus* spp. Further, *Ergasilus* spp. were confirmed as *E. sieboldi* by molecular characterization based on 28 S rRNA gene sequencing.

All forty-five diseased gilthead sea bream were infected with *E. sieboldi*. Therefore, the prevalence of *E. sieboldi* infestation was high (100%) in the gills, no parasites were found in any other organs (fin, skin, operculum, and other microhabitats) of the forty-five-gilthead sea bream. The mean infection intensity of *E. sieboldi* adult parasitic female in the gill filaments of gilthead sea bream was 32.4 ± 1.29 per fish gills. Infection intensity of copepod in the four gill arches of gilthead sea bream revealed that the highest infection intensity in the third gill arch (GA III) followed by the second gill arch (GA II), while the fourth gill arch (GA IV) showed the lowest infection intensity with *E. sieboldi* (Supplementary Table 1).

### Water quality parameters

The values of physicochemical parameters of water in the culture pond were temperature (30 ± 3 °C), pH (8.2 ± 0.2), dissolved oxygen (5 ± 0.5 mg/L), salinity (38 ± 2 PSU), ammonium-nitrogen (2.5 ± 0.3 mg/L), un-ionized toxic ammonia (0.37 ± 0.1 mg/L), nitrate (6 ± 3 mg/L), nitrite (0.09 ± 0.02 mg/L) and iron (0.40 ± 0.1 mg/L).

### Morpho-biochemical bacterial characterization

All isolates were gram-negative, curved rod shaped, and motile. They formed large yellow colonies on TCBS agar (sucrose fermenter isolates) and were positive for cytochrome oxidase, indole, and catalase tests. They showed swarming growth on blood agar and TSA. All the isolates were tolerant to high NaCl concentrations (up to 10%) and showed a similar carbohydrate fermentation profile. All isolates were confirmed as *V. alginolyticus* (99.5–99.9%) by API® (Table 1). Pathogenic *V. alginolyticus* were isolated from skin ulcer, muscle, and kidney. By contrast, no pathogenic *V. alginolyticus* were isolated from gill tissues.

**Table 1:** Biochemical characteristics of *V. alginolyticus* isolates retrieved from gilthead sea bream

Biochemical Tests	Results
Growth on TCBS	Large yellow colony
Swarming on blood agar and TSA	+
Growth at 28 C	+
Growth at 37 C	+
Growth at 0% NaCl	-
Growth at 2% NaCl	+
Growth at 10% NaCl	+
Gram stain	Gram -ve short rods
Motility on soft agar	+
Cytochrome oxidase	+
Catalase	+
Indole	+
Voges-Proskauer (VP)	-
Citrate utilization	-
Methyl red (MR)	+
Urea hydrolysis	-
Triple sugar iron (TSI)	K/A – A/A
Carbohydrate fermentation:	
Sucrose	+
Glucose	+
Lactose	-
Mannose	+
Mannitol	+
Sorbitol	-
Arabinose	-
Maltose	+
Rhammose	-
Melibiose	-
Inositol	-
Nitrate reduction	+
Hydrogen sulfide (H_2_S)	-
Esculin hydrolysis	-
Gelatin hydrolysis	-
Arginine dihydrolase	-
Ornithine decarboxylase	+
Lysine decarboxylase	+
Tryptophane production	+

+: positive; -: negative; K/A: alkaline slant / acid butt; A/A: acid slant / acid butt

### Molecular identification of parasite specimen and bacterial isolate

All forty-five diseased gilthead sea bream were co-infected with *E. sieboldi* and *V. alginolyticus. Ergasilus sieboldi* and *V. alginolyticus* were confirmed by molecular characterization based on 28 S rRNA and 16 S rRNA gene sequencing analysis, respectively. *Ergasilus sieboldi* and *V. alginolyticus* isolates co-infecting gilthead sea bream were sequenced, and the nucleotide sequences of the 28 S rRNA produced a total length of 688 bp, and 16 S rRNA produced a total length of 981 bp. Both nucleotides’ sequences were deposited in the GenBank database under accession numbers ON706996 (*E. sieboldi*) and ON041091 *(V. alginolyticus).* ON706996 was homologous with *E. Sieboldi* (MW810242 and OM812074), and the range of identity was 98–99% with an E value of 0.00. While ON041091 shared more than 99% identity (E value of 0.00.) with the accession numbers of *V. alginolyticus* (MN733128, MT368033, MH169304, OM654367, HQ827779 and KC884627).

### Virulence genes of *Vibrio alginolyticus* isolates

**Fig. 2:**
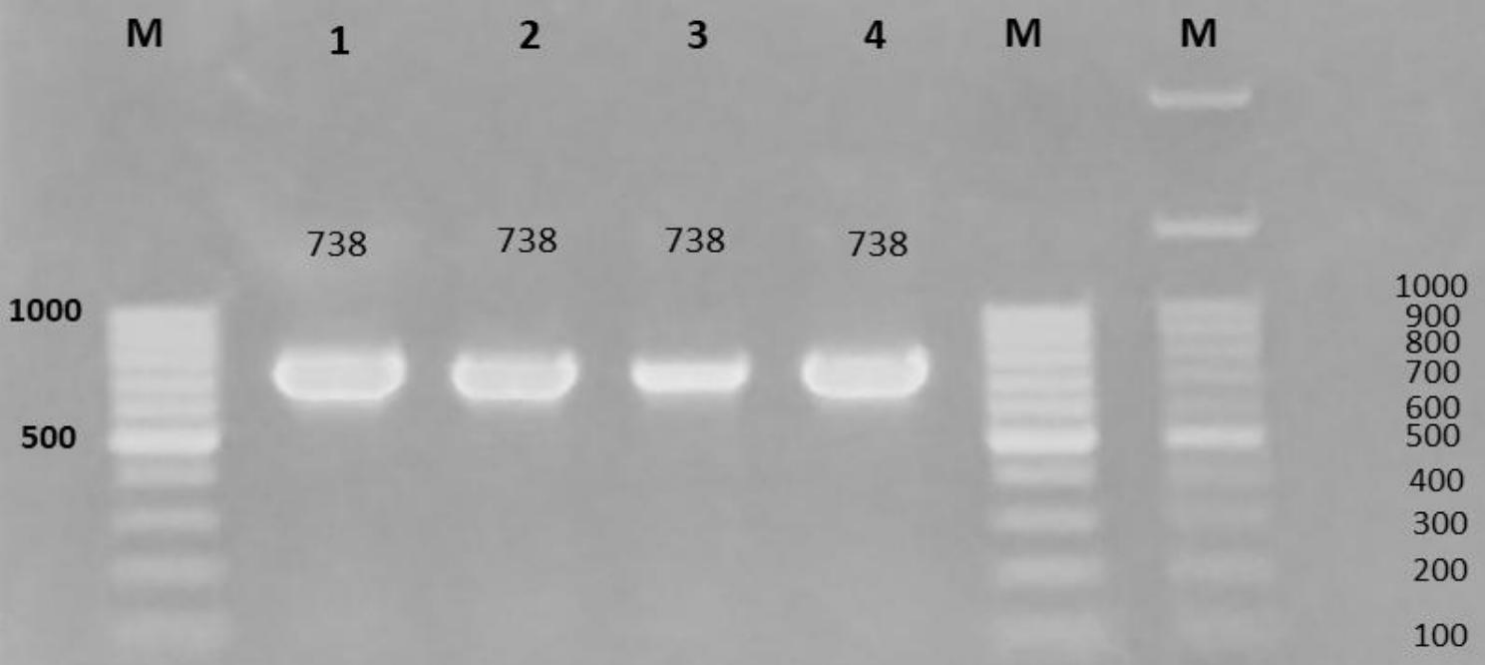
*Vibrio alginolyticus* virulence genes (*collagenase*, *VptoxR*, *tdh*) using Multiplex PCR. Agarose gel electrophoresis (1.5% agarose) of the *collagenase* (lanes 1–4) amplification products (738 bp) of *V. alginolyticus* strains. Lane: M, molecular weight marker. Neither amplification products (296 bp) for *VptoxR*, nor amplification products (270 bp) for *tdh*. Lanes 1, 2, 3, and 4 refer to VAK_4_, VASk_3_, VAM_6_, and VASp_1_ isolates, respectively

Multiplex PCR amplification was employed to detect *collagenase*, *VptoxR*, and *tdh* virulence genes in *V. alginolyticus* isolates. A 738 bp amplicon fragment matching *collagenase* was obtained in all *V. alginolyticus* strains whereas no bands were observed for *VptoxR* (296 bp) and *tdh* (270 bp) virulence genes (Fig. 2).

### Antimicrobial susceptibility test and detection of florfenicol resistance gene in *V. alginolyticus* isolates

Antimicrobial susceptibility tests revealed that all *V. alginolyticus* isolates were resistant to ampicillin (AMP 10) and erythromycin (E 15). Apart from ampicillin and erythromycin, 50% of isolates were resistant for trimethoprim/sulfamethoxazole (SXT 25) and novobiocin (NV 30). Regarding tetracyclines group, a considerable proportion of antimicrobial resistance to oxytetracycline (OT 30) and doxycycline (DO 30) was detected in this study. In the present study, only two isolates were susceptible to tetracyclines, while all isolates were sensitive to florfenicol (FFC 30). The multiple antibiotic resistance (MAR) index of *V. alginolyticus* ranged from 0.286 to 0.857 (Supplementary Table 2). The florfenicol resistant gene (*florR*) was not detected in all *V. alginolyticus* isolates.

### Oxidant/ antioxidant biomarkers in infected gilthead sea bream

**Fig. 3:**
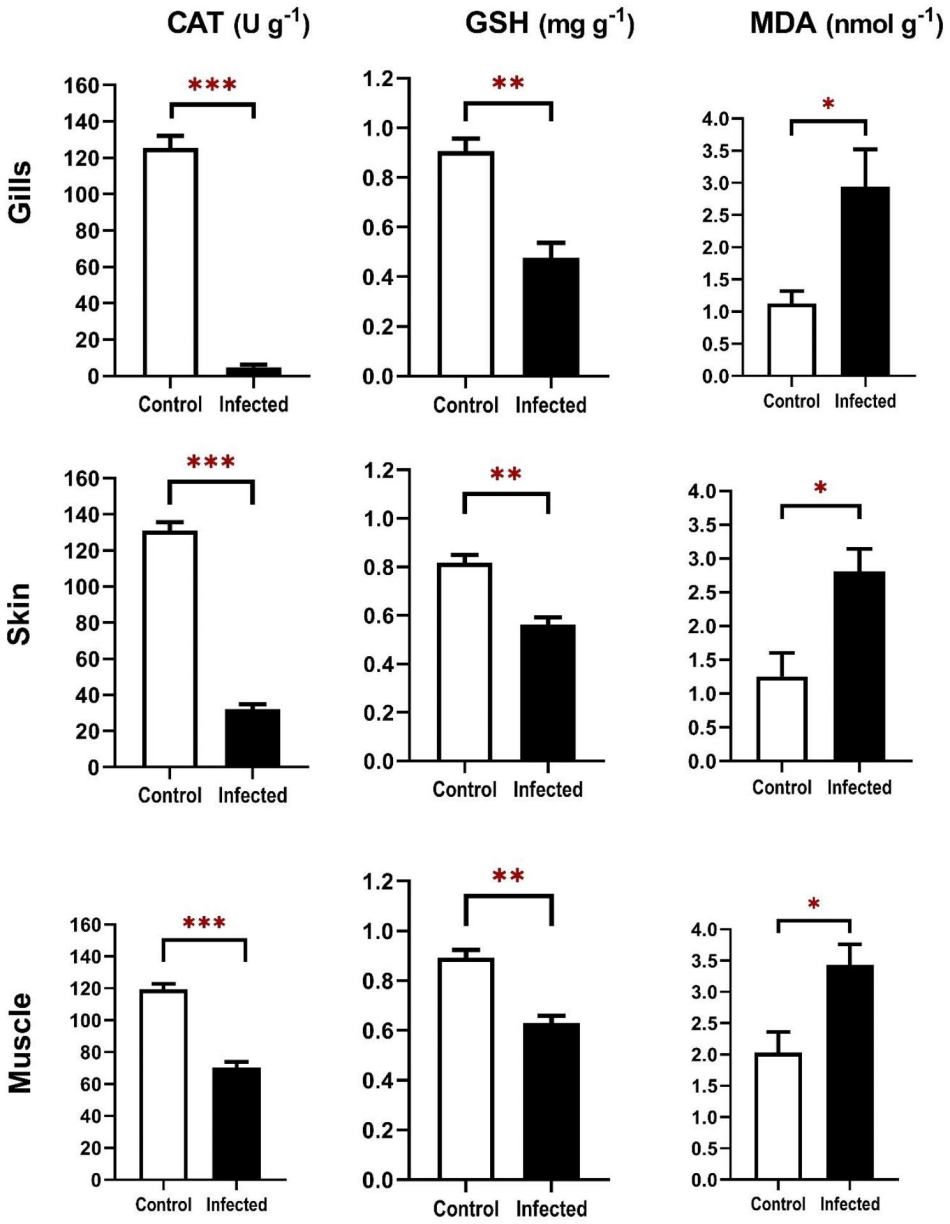
Malondialdehyde (MDA), catalase (CAT) and reduced glutathione (GSH) levels in gilthead sea bream naturally co-infected with *E. sieboldi* and *V. alginolyticus* analyzed in the gills, skin, and muscle tissues. White bar represents uninfected control fish, while black bar represents infected fish. The bars represent the mean ± standard error of the mean. Values are statistically significant at *p* value < 0.05 (Independent sample T-test, R 4.1.2). * (*p* value < 0.05), ** (*p* value < 0.01) and *** (*p* value < 0.001)

The activity of antioxidant enzymes (CAT and GSH) in the gills, skin and muscles of gilthead sea bream are shown in Fig. 3. Catalase and reduced glutathione antioxidant enzymes were decreased in the gills, skin, and muscles of gilthead sea bream co-infected with *E. sieboldi* and *V. alginolyticus* compared with uninfected control fish. The gills of diseased gilthead sea bream had the lowest levels of catalase and reduced glutathione antioxidant enzymes, followed by the skin and muscle. On the other hand, gilthead sea bream co-infected with *E. sieboldi* and *V. alginolyticus* showed a significant increase in Malondialdehyde (MDA) as a lipid peroxidation level (LPO) biomarker in the muscle, gills and skin compared with uninfected control fish. The muscle of diseased gilthead sea bream had the highest lipid peroxidation level followed by gills and skin (Fig. 3).

### Gene expression analysis of *cyp1a1*, *il-1β* and *tnf-α* in infected gilthead sea bream

**Fig. 4:**
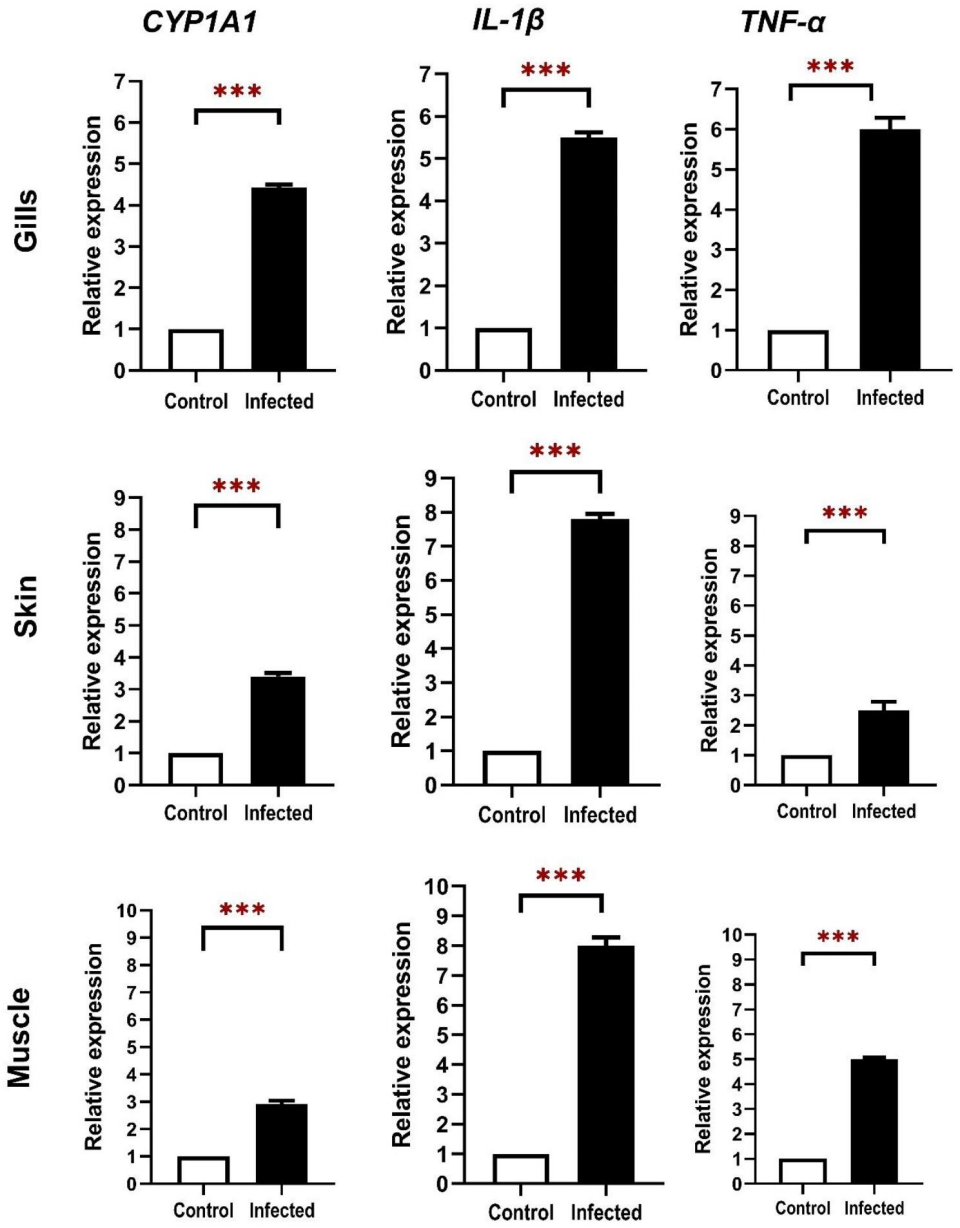
Fold changes comparing infected vs. uninfected control gilthead sea bream are shown. Relative expression of cytochrome P450 family 1 subfamily A member 1 (*cyp1a1*), interleukin-1β *(il-1β)* and tumor necrosis factor alpha (*tnf-α)* in gilthead sea bream naturally co-infected with *E. sieboldi* and *V. alginolyticus* analyzed in the gills, skin, and muscle tissues. The bars represent the mean ± standard error of the mean. Values are statistically significant at p value < 0.05 (Independent sample T-test, R 4.1.2). * (p value < 0.05), ** (p value < 0.01) and *** (p value < 0.001)

Significant up-regulation of *tnf-α*, *cyp1a1*, and *il-1β* expressions were detected in the gills of gilthead sea bream infected with *E. sieboldi* compared with uninfected control fish. On the other hand, *il-1β*, *cyp1a1*, and *tnf-α* expressions were up-regulated in the skin and muscle of gilthead sea bream infected with *V. alginolyticus* compared with uninfected control fish (Fig. 4).

### Histopathology

**Fig. 5:**
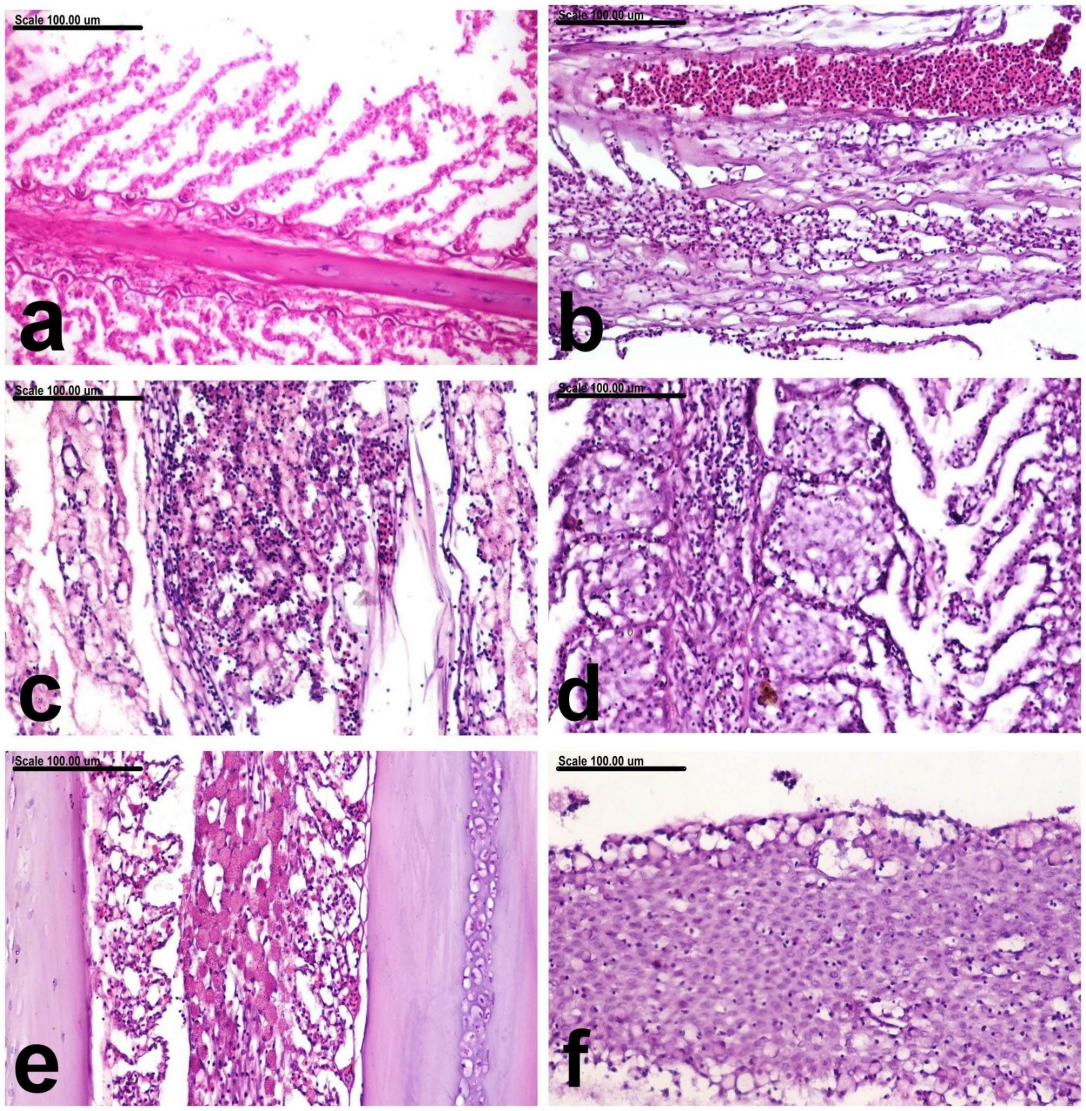
Photomicrograph representing histological sections from the gills of, **(a)** normal gills of uninfected control fish showing normal lamellar epithelium, with no evidence of proliferative or inflammatory reaction, **(b to f)** infested gilthead sea beam showing congestion of the venous sinuses and blood vessels associated with marked expansion of the primary gill lamellae with edematous fluid and inflammatory mononuclear cell infiltration **(b)**, congestion of the blood vessels with intense infiltration of the primary and secondary gill lamellae with mononuclear cells in addition to accumulation faint bluish of mucous exudate **(c)**, focal epithelial proliferation with focal epithelial fusion **(d)**, Mucous cell hyperplasia **(e)**, and hyperplasia of respiratory epithelium with complete fusion of secondary gill lamellae. The proliferating cells appeared large with round vesicular basophilic nuclei **(f)**. (Stain: H and E; Scale bar: 100 μm)

The gills of uninfected control fish showed normal lamellar epithelium, with no evidence of proliferative or inflammatory reactions (Fig. 5a). By contrast, the gill filaments of infected gilthead sea bream revealed various histopathological observations represented by severe congestion of the venous sinuses and blood vessels associated with marked expansion of the primary gill lamellae with edematous fluid and inflammatory mononuclear cell infiltration (Fig. 5b). Acute inflammatory reaction was a frequent lesion in the gill filament, characterized by congestion of the blood vessels with intense infiltration of the primary and secondary gill lamellae with mononuclear cells in addition to the accumulation of faint bluish mucous exudate (Fig. 5c). Focal epithelial proliferation with focal epithelial fusion was demonstrated in some gill lamellae (Fig. 5d). Mucous cell hyperplasia was also demonstrated (Fig. 5e). Hyperplasia of respiratory epithelium with complete fusion of secondary gill lamellae. The proliferating cells appeared large with round vesicular basophilic nuclei (Fig. 5f).

**Fig. 6:**
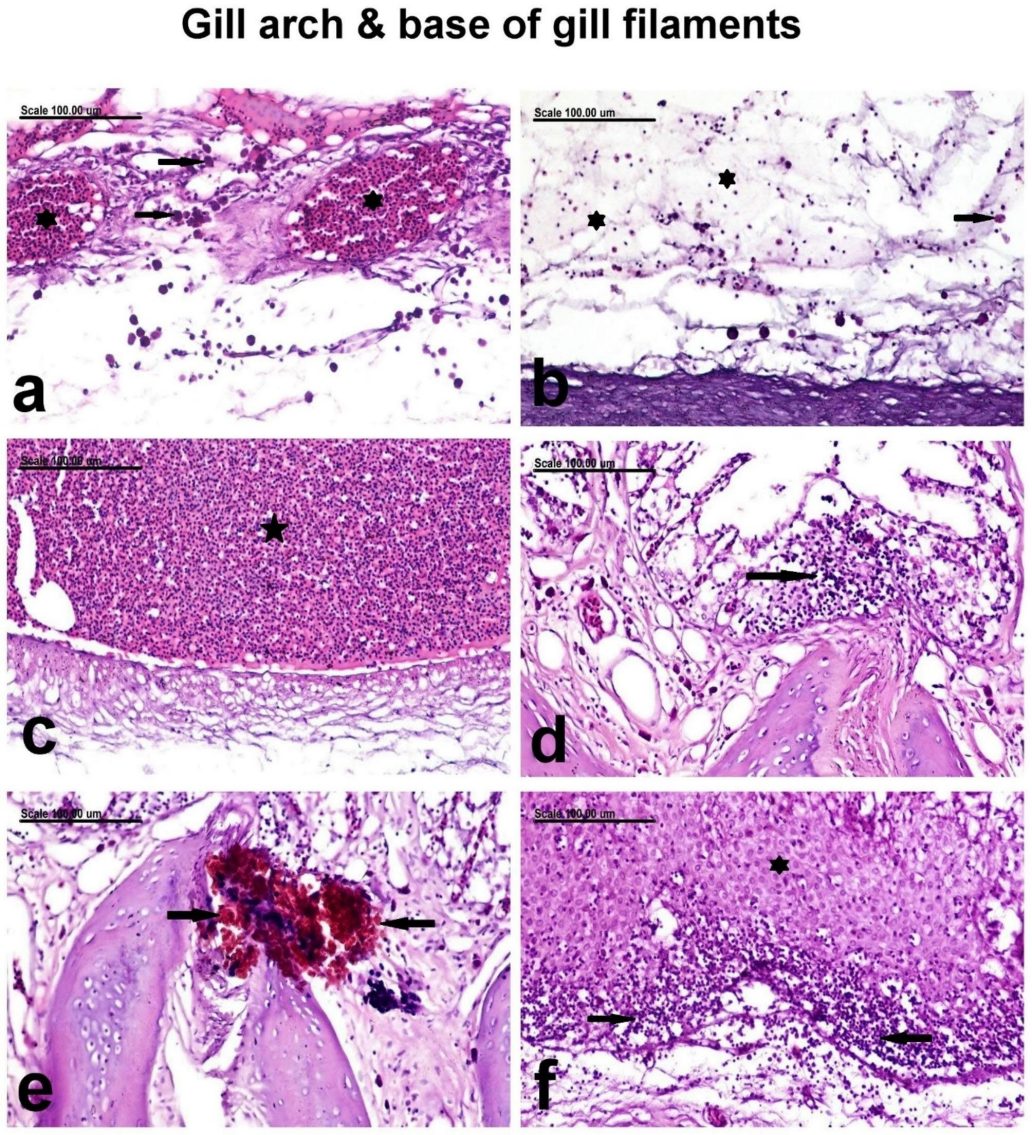
Photomicrograph representing histological sections from the gill arch and base of gill filaments of infected gilthead sea beam showing **(a)** severe congestion of the blood vessels (asterisk) associated with EGCs (arrows) and mononuclear cell infiltration, **(b)** extensive edema (asterisk) and infiltration of eosinophilic granular cells (EGCs) and lymphocytes (arrow), **(c)** massive hemorrhage (star), **(d)** focal infiltration of the epithelial lining the base of the gill filament with mononuclear cells (arrow), **(e)** focal infiltration of melanomacrophages (arrows), and **(f)** severe epithelial hyperplasia of the base of the gill filament (asterisk) concurrently with intense mononuclear cell infiltration (arrows). (Stain: H and E; Scale bar: 100 μm)

Variable histopathological observations in the gill arch of infected gilthead sea bream were demonstrated in Fig. 6. The gill arch showed severe congestion of the blood vessels (Fig. 6a) associated with extensive edema and infiltration of inflammatory cells, mainly eosinophilic granular cells (EGCs) and lymphocytes (Fig. 6b), in addition to massive hemorrhage (Fig. 6c). The base of the gill filament revealed congestion of the blood vessels associated with edema and multi-focal infiltration of their epithelial lining with mononuclear cells (Fig. 6d) and melanomacrophages (Fig. 6e). Hyperplastic proliferation of the epithelial cells lining the base of gill filaments concurrently with intense infiltration of the hyperplastic epithelium with mononuclear cells was frequently demonstrated (Fig. 6f). The hyperplastic epithelial cells appeared hypertrophied with large round vesicular basophilic nuclei (Fig. 6f).

**Fig. 7:**
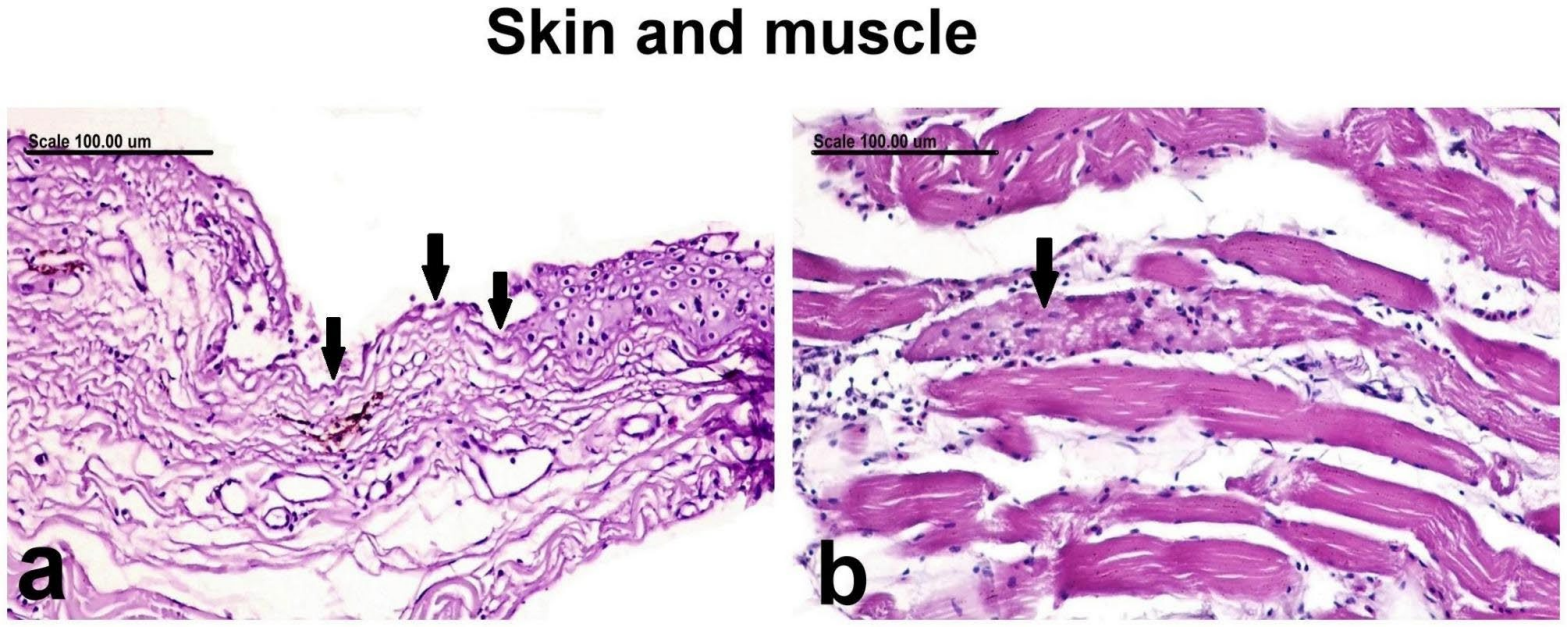
Photomicrograph representing histological sections from skin and muscle of infected gilthead sea beam showing **(a)** skin with focal erosive lesion with intact basement membrane (arrows), and **(b)** muscle with focal necrosis of individual myocyte which was infiltrated by mononuclear cells (arrow) (Stain: H and E; Scale bar: 100 μm)

No pronounced severe lesions were demonstrated in the skin of gilthead sea bream except for the focal erosive area with focal desquamation of the superficial epidermal cell layer, leaving an intact basement membrane (Fig. 7a). The underlying muscle appeared normal in most examined sections except for focal necrosis of individual myocytes, which is infiltrated by mononuclear cells (Fig. 7b).

## Discussion

Fish co-infected with parasitic and bacterial infections induce synergistic interaction resulting in severe illness and significantly higher mortalities [46]. The current study aimed to evaluate the oxidative stress, histopathology and gene expression of cultured gilthead sea bream co-infected with *V. alginolyticus* and *E. sieboldi* during the onset of summer mortalities.

In this study, the obtained behavioral and clinical signs of cultured gilthead sea bream were abnormal swimming behavior, evidence of asphyxia, and skin ulceration with fish mortalities. These clinical findings were described in previous studies [3, 4]. Further microscopic examination of gills revealed adult female *Ergasilus* species with elongated egg sacs embedded in the mucous exudates of the gill filament. Similar findings were recently described in gills of infected gilthead sea bream [47]. The insertion of the copepod antennae deep into gill tissue causes disruption of the gill filaments, results in severe respiratory problems in fish, especially during warms months [32, 33, 35]. Adult *E. sieboldi* were confirmed by 28 S rRNA sequencing, which produced a total length of 688 bp amplicon. It was found that the morphometric characteristics of *E. sieboldi* were similar to those described in previous studies [7, 47–50]. *Ergasilus sieboldi* is one of the most widespread ergasilid species that has been reported from fifteen fish families in Europe, Asia, and Africa [47, 48, 51]. The molecular data available for *E. sieboldi* includes species of African, Asian, and European origin [47, 48, 52, 53]. The analysis of the 28 S rRNA gene sequence of *E. sieboldi* (Accession number: ON706996) revealed identity (99%) with *E. sieboldi* of European origin (Accession number: MW810242) [48] and African origin (Accession number: OM812074) [47].

Typical signs of *V. alginolyticus* infection in gilthead sea bream were described in earlier studies [5, 13, 54]. Further, *V. alginolyticus* strains were confirmed by 16 S rRNA sequencing and revealed more than 99% identity with the *V. alginolyticus* strains deposited previously in NCBI.

In this study, forty-five cultured gilthead sea bream fish were co-infected with *E. sieboldi* and *V. alginolyticus*. No parasites were detected in the fish skin, fin, operculum, mouth, and other microhabitats. Co-infection with bacteria and parasites is a common occurrence in aquaculture. Infections with parasites not only increase the risk of secondary bacterial diseases but also have the potential to function as a vehicle for the transmission of disease-causing bacteria [17]. The majority of ergasilids infest the gills of their hosts are rarely found on the skin or other tissues [55]. The prevalence and infection intensity of *E. sieboldi* copepods in the gills of naturally infected gilthead sea bream was high (100%) and (32.4 ± 1.29) per fish gills, respectively. The copepods *Ergasilus* spp. in the gills of *Scatophagus argus* showed the highest prevalence (78.6%) and intensity [17.8 (1–233)] [56].

Physiological stress and physical injury are the primary contributing factors of fish disease and mortality in aquaculture [57, 58]. In this study, high water temperatures (30 ± 3 °C), salinity (38 ± 2 PSU), non-ionized toxic ammonia (0.37 ± 0.1 mg/L) and iron (0.40 ± 0.1 mg/L) were found to constitute likely stress factors resulting in increased fish susceptibility to infections [5, 25]. Poor water quality in fishponds was linked with mortalities of cultured fish due to parasitic and bacterial co-infections [59]. Temperature can also influence the severity of co-infection in fish by affecting the activity of the innate immune system. An increase in temperature accelerates co-infection intensity and adversely affects immunological and physiological parameters [60]. Parasites readily respond to changes in temperature. Commonly, increases in temperature can boost parasite reproduction, accelerate and shorten their life cycles and extend their transmission periods [61]. The high intensity of *E. sieboldi* in gills of cultured gilthead sea bream could be attributed to high water temperature in late summer, considering that rates of oviposition and egg hatching of *Ergasilus* spp. are greater at higher temperature [62]. At water temperature above 23 °C, the rates of oviposition and egg hatching of *Ergasilus* spp. are increased, thus affecting availability of copepod’s infective stages [63, 64]. Salinity is considered one of the main factors influencing the infection with ectoparasitic copepods. Ergasilid copepods are euryhaline metazoan ectoparasites which can tolerate a wider range of (19.7–31.2 PSU) [56]. *Ergasilus labracis* are an estuarine or freshwater parasite that are indeed tolerant of a wide range of salinities (10.2–30.2 PSU) [65]. In contrast, *E. labracis* was found to be the most prevalent parasitic copepod in fish reared in low to moderate salinities ranging from 13.56 to 21.11 PSU, and rarely on fish reared in high-salinity zone (more than 29 PSU). Moreover, parasite intensities were highest in the low-salinity zone and decreased significantly in high salinity zone [66].

*Vibrio* species are ubiquitous inhabitants of aquatic environments including estuaries, marine coastal waters, sediments and aquaculture facilities [67]. Currently, genus *Vibrio* comprises more than 130 species grouped in fourteen clades [68]. PCR-based approaches of *Vibrio* species targeting species-specific virulence genes have proven useful in discerning between closely related *Vibrio* species [69]. Several *V. alginolyticus* and *V. parahemolyticus* molecular protocols have been established based on the detection of collagenase and hemolysin encoding genes [70, 71]. PCR targeting species-specific virulence genes is an efficient approach to accurately determine the presence of opportunistic/pathogenic bacteria in complex microbial communities inhabiting aquaculture facilities [69]. *Vibrio alginolyticus* screening for the presence of virulence genes (*collagenase*, *VptoxR*, and *tdh*) revealed that all isolates produce only a 738-bp amplicon fragment matching collagenase gene, while *VptoxR* and *tdh* genes were negative in all tested strains. *Vibrio alginolyticus* strains had a MAR index ranging from 0.286 to 0.857. It was higher than findings from earlier studies in Egyptian aquaculture [5] or in different areas [72, 73]. A considerable proportion of antimicrobial resistance to oxytetracycline and doxycycline might be attributed to the frequent application of tetracyclines to control vibriosis in Egyptian marine fish farms during the past decade [5].

In the present study, a significant decrease in the activity of the antioxidant enzymes (CAT and GSH) as well as a significant increase in the activity of lipid peroxidation level were reported in the gills, skin, and muscle of gilthead sea bream infected with *E. sieboldi* and *V. alginolyticus* compared with uninfected control fish. The cellular damage of gills due to *E. sieboldi* leads to significant decrease in the activity of antioxidant enzymes in gills. Additionally, the cellular damage of skin and muscle tissues due to *V. alginolyticus* also leads to significant decrease in the activity of antioxidant enzymes in skin and muscle. Malondialdehyde is a byproduct of the peroxidative breakdown of polyenic fatty acids in the lipid peroxidation process, and its accumulation in tissues indicates the degree of free radical formation, lipid peroxidation, and oxidative stress [74]. Lipid peroxidation is essentially a toxic response to oxidative damage to cellular and tissue components [75]. The elevated levels of LPO affect the cell membrane permeability and mitochondrial oxidative phosphorylation, which could damage cellular membrane and stimulate cell apoptosis [76]. Oxidative stress is defined as the imbalance between oxidants and antioxidant defenses [77]. The infection leads to the imbalance between oxidant and antioxidant defense mechanisms through inhibiting the antioxidant activity as well as the inability to neutralize the impact of ROS released [25, 78, 79].

The innate immune system of fish is a vital defence mechanism against various pathogens. Co-infections in fish can elicit various alterations in the expression of immune genes in fish, which may include the up-regulation of genes associated with innate immunity. Up-regulation of *tnf-α*, *cyp1a1*, and *il-1β* expressions occurred in the three tissues of gilthead sea bream. Most inflammatory genes were up-regulated in the skin of Indian major carp, *Labeo rohita* in response to crustacean ectoparasite infection [80, 81]. *Il-1ß* is a proinflammatory cytokine with a significant role in the initial stage of inflammation by attracting fish leucocytes. Hence, up-regulation of *il-1ß* expression is a common response to microbial infections [45, 82, 83]. *Tnf-α* is an important mediator in response to parasitic, bacterial and viral infections [28, 41, 45]. Cytokines (*il-1β* and *tnf-α*) were up-regulated in kidney and brain of gilthead sea bream infected with nodavirus [84]. Significant up-regulation of *cyp1a1* was detected in the liver of gilthead sea bream reared in mixed sediments. *Cyp1a1* is activated by one specific heavy metal, or a consequence of exposure to all heavy metals existing in the sediments [85]. Up-regulation of *cyp1a1* expression was observed in channel catfish infected with *Edwardsiella ictaluri* infection [86]. Up-regulated *cyp1a1* expression is a common response to viral infection by reovirus in grass carp, *Ctenopharyngodon idella* [87]. This significant up-regulation in *cyp1a1*, *il-1ß*, and *tnf-α* expression might contribute to proinflammatory and inflammatory responses due to bacterial and parasitic infections [26, 44, 45, 88].

The skin and gills are important mucosal barriers that protect fish from the external environment. Fish mucosal surfaces harbor microbiome and mucosal immunity [89]. Mucosal surfaces can be colonized by commensals that provide benefits to the host by providing nutrients or protection from pathogens [90]. Homeostasis between the microbiome and mucosal immunity is crucial for the overall health of fish. Changes in the environment could affect the microbiome, thus altering mucosal immunity [91]. Dysbiosis in the commensal microbes can turn a commensal into a pathogen. Parasite-induced dysbiosis modulating host microbiota composition and host immune system [92]. For instance, this microbial dysbiosis can disrupt homeostasis on the mucosal surface, facilitating pathogen invasion and colonization by opportunist pathogenic bacteria (*Vibrio* spp.) from the surrounding environment [93]. Finally, parasites can directly carry new microbes that co-infect the host, and thus causing problems for fish [94].

Histopathological investigations of gilthead sea bream co-infected with *E. sieboldi* and *V. alginolyticus* revealed considerable damage and distortion of the normal tissue architecture. The extensive damage of gills were due to *E. sieboldi* copepods, while *V. alginolyticus* induces skin and muscle tissue reaction. Histopathological investigations of infected gills showed extensive tissue damage due to attachment and feeding of *E. sieboldi* copepods [3, 32]. *Ergasilus sieboldi* attaches to the outer surfaces of the gill filaments by powerful specialized antennae inserted deep into the gill tissues leading to severe mechanical injury, deformation of the gill filaments and puncturing of blood vessels [3]. Adhesions between gill filaments are shown as a response to copepods attachement. Consequently, fish respiration is impaired and reduced feeding, weight loss, and general deterioration of health can result [3, 4, 32]. The severity of damage caused by ergasilid infection of fish specimens is directly proportional to the number of copepods on the gills [86]. In the present study, the mean infection intensity of *E. sieboldi* copepods on the gills were low to moderate (32.4 ± 1.29) per fish gills. Accordingly, less than 20 *E. sieboldi* may have little effect, but, when their intensity exceeds 100, gill damage may be serious [95–97].

Histopathology of diseased fish with *Vibrio* spp. included sloughing of epithelial cells and severe necrotic muscle with massive infiltration of immune-related cells [98, 99]. Skin and muscle necrosis due to *V. alginolyticus* in gilthead sea bream was also reported [12].

## Conclusion

We have identified *E. sieboldi* and *V. alginolyticus* co-infections in gilthead sea bream cultured in a land-based farm. The prevalence of co-infections was 100%, no resistant fish were detected. *Ergasilus sieboldi* copepods infest the gill filaments of gilthead sea bream and are rarely found on the skin or other microhabitates. High water temperature and poor water quality accelerate the intensity of co-infections which imbalance the health status of the fish by hampering the oxidant-antioxidant mechanisms and proinflammatory/inflammatory immune genes to more detrimental side. Our results suggest that simultaneous screening for bacterial and parasitic pathogens should be considered. Also, we recommend conducting a screening for potential viral pathogens. These microbial co-infections may occur in farmed fish. Our findings provide valuable information on the relationship between fish immune systems and co-infections. Thus, maintaining good water quality and accurate diagnosis of diseases will aid in establishing effective management and treatment to control pathogens in gilthead sea bream aquaculture.

### Methods

#### Culture conditions, sampling and clinical signs

In late-Summer 2020, cultured gilthead sea bream was reared on a semi-intensive earthen pond-based marine fish farm, located in East of Al Tafrreah, Port Said, Egypt. The Fish seed (average: 5 g) were purchased from governmental hatchery. The stocking density of fish was 0.5–0.75 kg/m^3^. The fishpond was rectangular in shape (120 m x 60 m x 1.25 m) supplemented with two or three paddle wheel aerators. The fish were fed 4.0 mm pellets with 42% crude protein and 16% crude fat. The fish were fed about 1.8% of their total body weight. This feed ratio could be optimized depending on water temperature, water quality and fish size. At the onset of disease, the age of cultured fish ranged from 12 to 13 months. The first observation of behavioral signs was at the end of July. The fish were monitored for one month (August 2020), the fish exhibited signs of abnormal behavior, respiratory distress, and skin ulceration. The cumulative mortalities of diseased gilthead sea bream were moderate and ranged from 15 to 20%. The mortality pattern was irregular and ranged from a few dead fish to tens of dead fish daily.

To investigate the cause of respiratory distress and skin ulceration, forty-five naturally infected gilthead sea bream (average weight: 250 ± 50 g) were collected. Also, We sampled fifteen control un-infected gilthead sea bream (average weight: 250 ± 50 g) from uninfected pond of the same farm. These fish exhibited neither behavioral signs nor clinical signs. The clinically affected fish and uninfected control fish were immediately placed in separate plastic bags containing water, under conditions of artificial aeration, and transported alive to the Aquatic Animal Medicine and Management Department, Faculty of Veterinary Medicine, Cairo University, where they were measured, euthanized with overdose of benzocaine (0.2 g/L). The clinical, parasitological, and microbiological examination were conducted on the euthanized fish.

#### Water quality parameters

The physicochemical parameters of the water were measured two times daily (at 9 am and 4pm) using multi-parameter portable measuring instrument (HI-9829, Hanna Instruments Inc., Romania). Briefly, dissolved oxygen (DO, ppm) was measured using HI-7609829-2 probe, while water temperature (°C), salinity (PSU) and pH were measured using HI-7609829-1 probe. Moreover, ammonium-nitrogen (NH_4_-N, ppm) and nitrate-nitrogen (NO_3_-N, ppm) were measured weekly (at 9 am) using HI-7609829-10 and HI-7609829-12 probes, respectively. Nitrite (NO_2_^−^, ppm) and total iron (ppm) were measured monthly (at 9 am) using HI-3873 nitrite test kit and HI-3834 iron test kit according to manufacturer’s protocol (Hanna Instruments Inc., Romania).

#### Parasitological examination

Skin, gills and muscles of naturally infected fish and uninfected control fish were carefully examined by naked eye and handheld lens for macroscopic parasites and then dissected under a dissecting microscope. Parasitic copepods were collected, cleaned and then preserved in 70% ethanol. Permanent slide preparations were made using the phenol-balsam method [100]. Different structures of the collected copepods were carefully observed to be identified [7]. Total body length of the collected copepods was measured. Body measurements were demonstrated by millimeters (mm) and provided as a mean value followed by the minimum and maximum values. The total number of copepods collected from gills of each fish were counted; the parasitic prevalence and mean intensity were calculated [101].

#### Bacterial isolation and characterization

Under complete aseptic conditions, Loopfuls from skin, muscles, gills and kidney of naturally infected fish and uninfected control fish were streaked onto Tryptic soy agar supplemented with 2% NaCl (TSA, Lab M, UK), blood agar (Oxoid) supplemented with (5% sheep blood, 2% NaCl), motility agar (TSB + 0.3% agar + 2% NaCl) and TCBS agar (Oxoid). Inoculated plates were incubated for 48–72 h at 28 °C and 37 °C. Pure colonies were re-streaked onto TSA + 2% NaCl for phenotypic and biochemical identification. Presumptive identification was accomplished using different phenotypic and biochemical tests (APIID Test Strips®, APIWEB™, Biomérieux, USA) following the manufacture instructions. Presumptively identified Pure cultures were stored at − 20 °C in tryptic soy broth (TSB) (Lab M) supplemented with 2% NaCl (Lab M) and 16% glycerol (Sigma-Aldrich) for further characterization.

#### DNA extraction, amplification, and sequencing

The genomic DNA of parasite specimen and bacterial isolates was extracted using a QIAamp DNA Mini Kit (Qiagen, GmbH, Hilden, Germany) following the manufacturer’s instructions. The 28 S rRNA and 16 S rRNA fragment was amplified using the 28 S and 16 S primers (Supplementary Table 3). PCRs were performed in a final volume of twenty-five µL, containing 2 µL of DNA (50 ng/ µL) template,12.5 µL of 2x DreamTaq® Green Master Mix (Thermo Fisher Scientific) and 0.5 µL (10 nmol L^− 1)^ of each primer. The PCR amplifications were performed using a MyCycler™ thermal cycler (Bio-Rad, USA) with the following cycling conditions according to [53] (Supplementary Table 4). The amplified products were analyzed by electrophoresis (100 V, 400 mA, and 60 min) on a 1.5% (W/V) agarose gel.

The PCR products were purified with a QIA quick PCR purification column (Qiagen, GmbH, Hilden, Germany) following the manufacturer’s instructions and then sequenced with an ABI 3730XL DNA sequencer (Biosystems™, USA). The sequences obtained were analysed using Sequencer software and newly generated sequences were searched against the NCBI using blastn (BLAST^®^) and deposited in GenBank under the following accession numbers: ON041091 for 16 S rRNA and ON706996 for 28 S rRNA.

#### Antimicrobial susceptibility testing

Antimicrobial susceptibility testing of *V. alginolyticus* isolates was performed using the standard disc diffusion method [102], using the fellowing antimicrobial discs : ampicillin (AMP 10), erythromycin (E 15), novobiocin (NV 30), florfenicol (FFC 30), oxytetracycline (OT 30), doxycycline (DO 30), and trimethoprim/sulfamethoxazole (SXT 25). The diameters of inhibition zones (mm) were measured and then interpreted according to previously published guidelines [103].

#### Detection of the virulence genes

The detection of *V. alginolyticus* virulence genes; *collagenase*, *VptoxR*, *tdh* gene (Supplementary Table 3) were performed using a Multiplex PCR. PCRs were performed in a final volume of twenty-five µL, containing 2 µL of DNA (50 ng/ µL) template,12.5 µL of 2x DreamTaq® Green Master Mix (ThermoFisher Scientific) and 0.5 µL (10 nmol L^− 1)^ of each primer. The PCR amplifications were performed using a MyCycler™ thermal cycler (Bio-Rad, USA) with the following cycling conditions according to [71] (Supplementary Table 4). The template-free reactions were included in the PCR setup as negative controls. The amplified products were analyzed by electrophoresis on a 1.5% (W/V) agarose gel.

#### Detection of florfenicol resistance gene

The detection of *V. alginolyticus* florfenicol resistant gene; *florR* gene [104] (Supplementary Table 3) was performed using PCR in a final volume of 25 µL, containing 1 µL of DNA (50 ng/ µL) template,12.5 µL of 2x DreamTaq® Green Master Mix (Thermo Fisher Scientific) and 0.5 µL (10 nmol L^− 1)^ of each primer. The PCR amplifications were performed using a MyCycler™ thermal cycler (Bio-Rad, USA) with the following cycling conditions according to [104] (Supplementary Table 4). The amplified products were analyzed by electrophoresis on a 1.5% (W/V) agarose gel.

#### Oxidant/ antioxidant biomarkers

Gills, skin and muscle tissues from naturally infected fish and uninfected control fish were homogenized in ice-cold 0.1 M phosphate buffer saline (pH 7.4) using a Teflon tissue homogenizer. The crude tissue homogenate was centrifuged at 4000 rpm for 15 min at 4^°^C, then the supernatants were stored at − 80 °C until use. Catalase activity (CAT) was measured using Catalase assay colorimetric method (Bio-diagnostic Co., Egypt) [105]. Malondialdehyde (MDA) as a lipid peroxidation level (LPO) biomarker was measured spectroscopically at 532 nm using the thiobarbituric acid method (Bio-diagnostic Co., Egypt) [106]. Reduced glutathione content (GSH) was measured using Reduced glutathione colorimetric method (Bio-diagnostic Co., Egypt) along with the manufacturer’s guides.

### Quantitative real-time PCR analysis of *cyp1a1* and of immune response genes (*il-1β* and *tnf-α*)

#### Sampling and RNA extraction

Gills, skin and muscle tissues from naturally infected fish and uninfected control fish were sampled and kept in RNA latter at 4 °C for 24 h and then stored at – 80 °C. Approximately, 30 mg of gill, skin, and muscle tissues was used for RNA extraction using total RNA Extraction Kit (Thermo Fisher Scientific, USA) following the manufacturer’s protocol. The RNA concentration (ng/µL) and quality (A260/A280 ratios were 1.8–2.0) was assessed using Nanodrop™ 2000 Spectrophotometer (Thermo Fisher Scientific, USA).

#### Primer design and validation

Primer pairs, GenBank accession number, amplicon size and primer efficiencies for the target genes (*cyp1a1, il-1β and tnf-α*) [85, 107] and reference gene (*gapdh*) [108] and were summarized (Supplementary Table 5). These primers were selected due to their higher efficiency and their sequences best matches the sequences of the reference and target genes.

#### Reverse transcription quantitative polymerase chain reaction

Complementary DNA (cDNA) was synthesized from 1 µg of RNA using M-MuLV Reverse Transcriptase (New England Biolabs® Inc., MA, USA) according to the manufacturer’s protocol. The expression of the nominated genes (Supplementary Table 5) was analyzed by real-time qPCR with Bio-Rad iCycler thermal cycler and MyiQrealtime PCR detection system (BIO-RAD, USA). The qPCR reaction mixtures were performed in a total volume of 10 µL, containing 5 µL of Maxima SYBR Green/ROX qPCR Master Mix (2X) (Cat. No. K0221, Thermo Fisher Scientific, USA), 0.5 µL of primers (10 mM each), 1.5 µL of cDNA template and 2.5 µL nuclease free water. The real-time qPCR conditions were performed according to [85, 107, 108] as follows: 95 °C for 5 min (initial denaturation) and then 40 cycles at 95 °C for 15 s, 60 °C for 20 s, and 72 °C for 15 s. A melting curve analysis was performed after the amplification to confirm the specificity of the PCR products, through one cycle of 95 °C for 15 s, 55 °C for 15 s, and 95 °C for 15 s. In all cases, each PCR was performed with triplicate samples. The specificity of the reactions was analyzed using samples without cDNA as negative controls (NTC). For each mRNA, target gene expression was normalized with reference gene in each sample. The gene expression was analyzed using the 2^−ΔΔCT^ method [109].

### Histopathological investigations

Pieces of gills, skin and muscle tissues were excised from naturally infected fish and uninfected control fish, then rinsed in physiological saline and fixed in aqueous fixative (10% formal saline) for 24 h to preserve the structure and chemical constituents of tissues and cells. The tissues were dehydrated in ethyl alcohol series of ascending concentrations, then embedded in molten paraffin wax and cooled to harden the wax. Tissue blocks were cut into 5 μm thick sections using a rotatory microtome and then mounted onto glass microscope slides. After clearing and rehydration, the tissue sections can be stained using Hematoxylin and Eosin (H and E) according to the methods of [110]. Ten sections of each tissue from each fish were examined by a light microscope for histopathological evaluation.

### Statistical analyses

Statistical analyses were performed using R program [111] and GraphPad Prism (8.2.0, 2019). The normality of residuals and heteroscedasticity of variances were assumed using Shapiro-Wilk test and Levene’s test. Infection intensity of copepod was expressed as mean ± *SEM* (One way ANOVA, Tukey post hoc.). Oxidant/antioxidant biomarkers were expressed as mean ± *SEM*, while genes expression was presented as mean of copies of each gene (fold change) ± *SEM* (Independent sample T-test). The significance level was set at a probability value of less than 0.05 (p ˂0.05).

### Accession numbers

*Ergasilus sieboldi* (GenBank accession number ON706996) and *Vibrio alginolyticus* (GenBank accession number ON041091).

### Electronic supplementary material

Below is the link to the electronic supplementary material.

Supplementary Material 1

## Data Availability

The datasets analysed during the current study are available in the GenBank database under the accession numbers: ON706996 and ON041091.
