# Supplementary material for: Oxidative stress, gene expression and histopathology of cultured gilthead sea bream (Sparus aurata) naturally co-infected with Ergasilus sieboldi and Vibrio alginolyticus
Source: BMC Vet Res. 2023 Dec 16;19:277. doi: 10.1186/s12917-023-03840-9 (PMC10724927; doi:10.1186/s12917-023-03840-9)
Supplement: Supplementary file 1 — Supplementary Material 1 [file 12917_2023_3840_MOESM1_ESM.docx]

**Supplementary data**

**Oxidative stress, gene expression and histopathology of cultured gilthead sea bream (*Sparus aurata)* naturally co-infected with *Ergasilus sieboldi* and *Vibrio alginolyticus***

**Mahmoud Abou-Okada ^1*^,** **Maha M. Rashad ^2^, Ghada E. Ali** **^2^, Shimaa Abdel-Radi ^3^, Azza Hassan ^4^**

^1^ Department of Aquatic Animal Medicine and Management, Faculty of Veterinary Medicine, Cairo University, Giza, 12211, Egypt.

^2^ Department of Biochemistry and Chemistry of Nutrition, Faculty of Veterinary Medicine, Cairo University, Giza 12211, Egypt.

^3^ Department of Parasitology, Faculty of Veterinary Medicine, Cairo University, Giza, 12211, Egypt.

^4^ Department of Pathology, Faculty of Veterinary Medicine, Cairo University, Giza, 12211, Egypt.

*Corresponding author, Department of Aquatic Animal Medicine and Management, Faculty of Veterinary Medicine, Cairo University, Giza, 12211, Egypt.

E-mail addresses: abouokada.mm@cu.edu.eg; mahmoud.mehani@gmail.com (Mahmoud Abou-Okada)

**Supplementary data Pages: 6**

**Supplementary data Tables: 5**

**References**

**Table S1.** The mean intensity of *Ergasilus sieboldi* infestation in the gill arches of naturally infected gilthead sea bream

| **Gill arch** | **Mean* ± SEM** | **F-value** | **P-value** |
| --- | --- | --- | --- |
| First gill arch (GA I) | 6.00 ± 0.707**^b^** | 21.059 | 0.0001 |
| Second gill arch (GA II) | 9.40 ± 0.748**^a^** |  |  |
| Third gill arch (GA III) | 12.20 ± 0.860**^a^** |  |  |
| Fourth gill arch (GA IV) | 4.80 ± 0.583**^b^** |  |  |
| Total (four gill arches) | 32.4 ± 1.29 |  |  |

Data are represented as mean ± *SEM*. Values are statistically significant at *p* value < .05 (One way ANOVA, Tukey post hoc., R 4.1.2). Columns with different superscript are statistically significant.

*The mean intensity of infestation in the gill arches of gilthead sea bream represents the adult parasitic female. The adult parasitic female of *E. sieboldi* was determined and counted based on the morphology of *E. sieboldi* described by [1, 2].

**Table S2.** Antimicrobial susceptibility testing using disc diffusion method and MAR index of *Vibrio alginolyticus* isolates.

| Antimicrobial agents | Isolate code | # Tested antimicrobials | # Antimicrobial resistance | MAR index |
| --- | --- | --- | --- | --- |
| AMP10, E15 | VAM6 | 7 | 2 | 0.286 |
| AMP10, E15, NV30, SXT25 | VASk3 | 7 | 4 | 0.571 |
| AMP10, E15, OT30, DO30 | VASp1 | 7 | 4 | 0.571 |
| AMP10, E15, NV30, SXT25, OT30, DO30 | VAK4 | 7 | 6 | 0.857 |

MAR index, multiple antibiotic resistance index; AMP, ampicillin; E, erythromycin; NV, novobiocin; SXT, sulfamethoxazole/trimethoprim; OT, oxytetracycline; DO, doxycycline; 10, 10 µg; 15, 15 µg; 30, 30 µg; 25, 25 µg; VAM, *Vibrio alginolyticus* isolated from muscle tissues of gilthead sea bream fish; VASK, *Vibrio alginolyticus* isolated from skin of gilthead sea bream fish; VASp, *Vibrio alginolyticus* isolated from spleen tissues of gilthead sea bream fish; VAK, *Vibrio alginolyticus* isolated from kidney tissues of gilthead sea bream fish. All tested isolates were susceptible to florfenicol (FFC 30).

**Table S3.** Primers used for PCR.

| Primer name | Sequence (5´-3´) | Amplicon size (bp) | References |
| --- | --- | --- | --- |
| 28S rRNA | F: ACA ACT GTG ATG CCC TTA G  R: TGG TCC GTG TTT CAA GAC G | 688 | [3] |
| 16S rRNA | F: CTG GAA CTG AGA CAC GGT CC  R: ACG CAC TTT TTG GGA TTC GC | 981 | This study* |
| *VptoxR* gene | F: GAG TTT GTT TGG CGT GAG CAA G  R: GGT TCA ACG ATT GCG TCA GAA G | 296 | This study* |
| Collagenase gene | F: CGA GTA CAG TCA CTT GAA AGC C  R: CAC AAC AGA ACT CGC GTT ACC | 738 | [4] |
| *tdh* gene | F: TGG AAT AGA ACC TTC ATC TTC ACC  R: GTA AAG GTC TCT GAC TTT TGG AC | 270 | [5] |
| *FlorR* gene | F: TCC TGA ACA CGA CGC CCG CTA T  R: TCA CCG CCA ATG TCC CGA CGA T | 960 | [6] |

* Primer design by PrimerQuest^®^ Tool.

F, forward primer; R, reverse primer; bp, base pair

**Table S4.** PCR cycling conditions of different genes in this study.

| **Gene** | **Initial denaturation** | **# Cycles** | **Denaturation** | **Annealing** | **Extension** | **Final extension** |
| --- | --- | --- | --- | --- | --- | --- |
| **18S rRNA** | 94°C / 5 min | 30 | 94°C / 30 s | 54°C / 30 s | 72°C / 60 s | 72°C / 5 min |
| **16S rRNA** | 95°C / 10 min | 30 | 94°C / 60 s | 55°C / 60 s | 72°C / 60 s | 72°C / 5 min |
| ***VptoxR*** | 95°C / 10 min | 35 | 94°C / 60 s | 58°C / 30 s | 72°C / 60 s | 72°C / 5 min |
| **Collagenase** |  |  |  |  |  |  |
| ***tdh*** |  |  |  |  |  |  |
| ***FlorR*** | 95°C / 5 min | 35 | 94°C / 30 s | 60°C / 60 s | 72°C / 60 s | 72°C / 5 min |

**Table S5.** Primers used for real-time qPCR.

| Gene name | Gene abbreviation | Primer sequence (5′-3′) | Primer size (bp) | Tm (°C) | GC% | E (%) | GenBank Acc. Num. | References |
| --- | --- | --- | --- | --- | --- | --- | --- | --- |
| Interleukin-1β | *il-1β* | F: GCG AGC AGA GGC ACT TAG TC | 20 | 60 | 60 | 103.5 | AJ277166 | [7] |
|  |  | R: GGT AGG TCG CCA TGT TCA GT | 20 |  | 55 |  |  |  |
| Tumor necrosis factor alpha | [*tnf-α*](https://www.sciencedirect.com/topics/agricultural-and-biological-sciences/tumor-necrosis-factor-alpha) | F: CTG TGG AGG GAA GAA TCG AG | 20 | 60 | 55 | 112.93 | AJ413189 | [7] |
|  |  | R: TCC ACT CCA CCT GGT CTT TC | 20 |  | 55 |  |  |  |
| Cytochrome P450, family 1, subfamily A, member 1 | *cyp1a1* | F: CGT GTT GAG TGG CAG TGA AA | 20 | 60 | 50 | 101 | AF011223 | [8] |
|  |  | R: CTT TGC TGA CGT GTT CCT CC | 20 |  | 55 |  |  |  |
| Glyceraldehyde-3-phosphate dehydrogenase | *gapdh* | F: CCA GCC AGA ACA TCA TCC | 18 | 60 | 55.5 | 103.5 | DQ641630 | [9] |
|  |  | R: GCA GCC TTG ACG ACC TTC | 18 |  | 61 |  |  |  |

F, forward primer; R, reverse primer; Tm, annealing temperature; bp, base pair; E, amplification efficiency; Acc. Num., accession number.

**References**

1. Abdelhalim AI, Lewis JW, Boxshall GA. The life-cycle of *Ergasilus sieboldi* Nordmann (Copepoda: Poecilostomatoida), parasitic on British freshwater fish. Journal of Natural History 1991; 25(3), 559-582. <https://doi.org/10.1080/00222939100770361>.
2. Boxshall GA., Halsey SH. An introduction to copepod diversity. Ray Society; 2004.
3. Song Y, Wang GT, Yao WJ, Gao Q, Nie P. Phylogeny of freshwater parasitic copepods in the Ergasilidae (Copepoda: Poecilostomatoida) based on 18S and 28S rDNA sequences, Parasitol. Res. 2008; 102, 299–306. <https://doi.org/10.1007/s00436-007-0764-8>.
4. Di Pinto A, Ciccarese G, Tantillo G, Catalano D, Forte VT. A collagenase-targeted multiplex PCR assay for identification of *Vibrio alginolyticus, Vibrio cholerae*, and *Vibrio parahaemolyticus*. J Food Prot. 2005; 68(1), 150-153. <https://doi.org/10.4315/0362-028x-68.1.150>.
5. Nishibuchi M, Kaper JB. Nucleotide sequence of the thermostable direct hemolysin gene of *Vibrio parahaemolyticus*. J Bacteriol. 1985; 162(2), 558-64. <https://doi.org/10.1128/jb.162.2.558-564>.
6. Dai L, Lu LM, Wu CM, Li BB, Huang SY, Wang SC, Qi YH, Shen JZ. Characterization of antimicrobial resistance among *Escherichia coli* isolates from chickens in China between 2001 and 2006. FEMS Microbiol Lett. 2008; 286, 178-183. <https://doi.org/10.1111/j.1574-6968.2008.01272.x>.
7. Campos-Sánchez JC, Mayor-Lafuente J, Guardiola FA, Esteban MÁ. In silico and gene expression analysis of the acute inflammatory response of gilthead seabream (*Sparus aurata*) after subcutaneous administration of carrageenin. Fish Physiol Biochem.2021; 47(5), 1623-1643. <https://doi.org/10.1007/s10695-021-00999-6>.
8. Benhamed S, Guardiola FA, Martínez S, Martínez-Sánchez MJ, Pérez-Sirvent C, Mars M, Esteban MÁ. Exposure of the gilthead seabream (*Sparus aurata*) to sediments contaminated with heavy metals down-regulates the gene expression of stress biomarkers. Toxicol Rep. 2016; 26(3), 364-372. <https://doi.org/10.1016/j.toxrep.2016.02.006>.
9. Salmerón C, Riera-Heredia N, Gutiérrez J, Navarro I, Capilla E. Adipogenic Gene Expression in Gilthead Sea Bream Mesenchymal Stem Cells from Different Origin. Front Endocrinol (Lausanne) 2016; 22(7), 113. <https://doi.org/10.3389/fendo.2016.00113>.
